# Programs for fixed-time artificial insemination in South American beef cattle

**DOI:** 10.21451/1984-3143-AR2018-0025

**Published:** 2018-08-03

**Authors:** Gabriel A. Bó, Emilio Huguenine, José Javier de la Mata, Richard Núñez-Olivera, Pietro S. Baruselli, Alejo Menchaca

**Affiliations:** 1 Instituto A.P. de Ciencias Básicas y Aplicadas, Carrera de Medicina Veterinaria, Universidad Nacional de Villa María, Córdoba, Argentina; 2 Instituto de Reproducción Animal Córdoba (IRAC), Zona Rural General Paz, 5145, Córdoba, Argentina; 3 Departamento de Reprodução Animal, FMVZ-USP, CEP 05508-000 São Paulo, Brazil; 4 Private Practice, SAV, San Luis, Argentina; 5 Facultad de Agronomía, Universidad Nacional de La Pampa, Santa Rosa, La Pampa, Argentina; 6 Fundación IRAUy, Instituto de Reproducción Animal Uruguay, Montevideo, Uruguay

**Keywords:** color-flow Doppler ultrasonography, proestrus length, sexed-sorted semen.

## Abstract

Fixed-time artificial insemination (FTAI) has been widely applied in South America within the last 20 years for the genetic improvement of commercial beef herds. Most FTAI treatments for beef cattle used in South America are based on the use of progesterone (P4) releasing devices and estradiol to synchronize follicle wave emergence, with pregnancies per AI (P/AI) ranging from 40 to 60%. More recent protocols focusing on extending the interval from device removal to FTAI (i.e. increasing the growing period of the ovulatory follicle) have been reported to improve P/AI in beef cattle. These new protocols and the more traditional FTAI protocols have also been adapted for use with sexed-sorted semen with acceptable P/AI in beef cattle. Finally, color-flow Doppler ultrasonography has been incorporated recently to determine the vascularity of the CL and thereby detect pregnancy as early as Day 22 after the first AI for re- synchronization of ovulation for a second FTAI in non- pregnant animals. In summary, FTAI protocols have facilitated the widespread application of AI in South American beef cattle by allowing for the insemination and re-insemination of herds during a defined breeding season, without the necessity of clean up bulls to achieve high pregnancy rates.

## Introduction

Artificial insemination (AI) is the most useful method for genetic improvement in cattle, and because estrus detection is difficult and inefficient, fixed-time artificial insemination (FTAI) is necessary. There are basically two types of FTAI protocols currently used in beef cattle; GnRH-based and estradiol-based protocols, both of which are combined with progesterone (P4) releasing devices and prostaglandin F_2α_ (PGF_2α_). Estradiol/P4-based protocols are most commonly used in South-American beef herds because of the availability of estradiol, its high effectiveness both in heifers and in cows in postpartum anestrus, and its relative low cost. With 20 years of experience with FTAI in South America, results are now more consistent with pregnancy per AI (P/AI) ranging between 40 to 60% (Bó *et al*., 2013). However, recently developed protocols that extend the period from P4 device removal to ovulation (defined as the proestrus period) have provided new opportunities for increasing P/AI (Bridges *et al*., 2008; Bó *et al*., 2016). Furthermore, treatments to re-synchronize ovulation have provided the opportunity to do sequential FTAI, without the necessity of using clean-up bulls (Baruselli *et al*., 2017a). The objective of this manuscript is to review protocols that are currently available and discuss their applications in beef herds.

## Estradiol/P4-based treatments for FTAI

Estradiol and P4 treatments consist of insertion of a P4 releasing device and the administration of 2 mg of estradiol benzoate (EB) on random days of the cycle (Day 0; to induce follicle atresia and synchronize follicular wave emergence), PGF_2α_ at the time of P4 device removal on Days 7, 8 or 9 (to ensure luteolysis) and the subsequent application of 1 mg EB 24 h later, GnRH or LH 54 h later or 0.5 or 1 mg of estradiol cypionate (ECP) at the time of P4 device removal (Bó *et al*., 2013) to synchronize ovulation. Most practitioners prefer the use of ECP to synchronize ovulation because it reduces the need to handle animals for the administration of EB. Treatment protocols that are applied to suckling beef cows usually include the administration of equine chorionic gonadotropin (eCG) at the time of removal the P4 device (Baruselli *et al*., 2004; Bó *et al*., 2013), which has been reported to stimulate the growth of the dominant follicle, increased ovulation rate (Sá Filho *et al*., 2010a; Núñez-Olivera *et al*., 2014) and circulating P4 concentrations in the subsequent luteal phase in cows experiencing postpartum anestrus (Baruselli *et al*., 2012; Núñez-Olivera *et al*., 2014). Although the use of eCG has been widely used in *Bos indicus* herds with high incidence of postpartum anestrus (Baruselli *et al*., 2004; Sá Filho *et al*., 2010a) improvements in *Bos taurus* herds with high incidence of postpartum anestrus have been also reported (Menchaca *et al*., 2013; Núñez-Olivera *et al*., 2014), while no improvements in P/AI has been reported in herds in with high proportions of cycling cows or heifers at the time of treatment (reviewed in Bó and Baruselli, 2014).

## GnRH-based treatments

GnRH-based protocols are used widely for beef cattle in North-America and Europe, but GnRH use is limited in South-America because of cost and the availability of estradiol esters. The most commonly used protocol is called Co-Synch; GnRH is administered at the time of FTAI to synchronize ovulation (Geary *et al*., 2001). In general Co-Synch protocols have included the insertion of a P4 device to overcome poor ovulation rates after the first GnRH in heifers (Martinez *et al*., 2002) and in postpartum suckled beef cows experiencing anestrus (Lamb *et al*., 2001). Data on the addition of eCG to GnRH/P4-based treatment protocols have been more controversial, with reported improvements in P/AI in *Bos indicus* (Pincinato *et al*., 2012) and *Bos taurus* cows in postpartum anestrus (Huguenine *et al*., 2013) and in primiparous *Bos taurus* cows that had not been pre-synchronized (Small *et al*., 2009). However, no improvement in P/AI has been reported in *Bos taurus* cows with low incidence of postpartum anestrus and moderate to high body condition scores (BCS; Marquezini *et al*., 2013).

## Protocols that prolong the proestrus period

### Extending the proestrus period in GnRH/P4-based protocols

New protocols for FTAI were developed to prolong the period from P4 device removal to ovulation with the objective of incrementing the period of preovulatory estradiol exposure and improving uterine function and early embryo development (Bridges *et al*., 2008; 2012). The protocol was named 5-day Co- Synch+P4 and resulted in higher P/AI than with the 7- day Co-Synch+P4 in beef cows (Bridges *et al*., 2008; Whittier *et al*., 2013). The main changes in this protocol was a reduced period of insertion of the P4 releasing device from 7 to 5 days, to avoid the adverse effects of persistent follicles on fertility of the cows not ovulating to the first GnRH, and to prolong the period from P4 device removal to the GnRH treatment to increase the exposure to circulating estradiol concentrations prior to ovulation (Bridges *et al*., 2014). Higher estradiol concentrations in the preovulatory period have been associated to an increased ability of the uterus to support conceptus development (Bridges *et al*., 2013, Binelli *et al*., 2014) and were also related to lower embryonic losses in the time period between maternal recognition of pregnancy and placental attachment (Madsen *et al*., 2015).

Because of the shorter interval between the first GnRH and induction of luteolysis in the 5-day Co- Synch+P4 protocol, a single administration of PGF_2α_ was not effective in inducing luteolysis in beef cows that had ovulated to the GnRH (Souto *et al*., 2009); two doses of PGF_2α_ 8 to 12 hours apart resulted in higher P/AI (Kasimamickam *et al*., 2009). In a large field trial with 2,465 postpartum beef cows, P/AI was greater (P < 0.05) in cows receiving 2 PGF_2α_ 8 h apart (55%) than those receiving only one PGF_2α_ (48%), with those receiving 2 PGF_2α_ at the same time being intermediate (51%; Bridges *et al*., 2012). Hence, double PGF_2α_ given 8 to 24 h apart seemed necessary to maximize fertility with the 5-day protocol. If farm conditions do not permit the extra handling, a double dose of PGF_2α_ given at device removal would be an acceptable alternative.

The 5-day Co-Synch+P4 protocol has also been investigated in *Bos indicus* cows in South America, with lower P/AI in suckled Nelore cows than those treated with the conventional 8-day estradiol/P4-based protocol (Ferraz Jr *et al*., 2016). An important difference was that 400 IU eCG was used in the estradiol/P4-based protocol but it was not used in the 5-day Co-Synch+P4 protocol. To confirm this notion, we have reported no differences in P/AI in cycling cows treated with the 5-day Co- Synch+P4 and the estradiol/P4-based protocol, but P/AI was higher in cows in postpartum anestrus that received 400 IU eCG at P4 device removal (5-day Co-Synch+P4: 46.3%, 120/259; estradiol/P4-based: 54.5%, 151/277) than in cows treated with 5-day Co-Synch+P4 but without eCG (26.8%, 71/265; P < 0.05; Huguenine *et al*., 2013).

**Figure 1:**
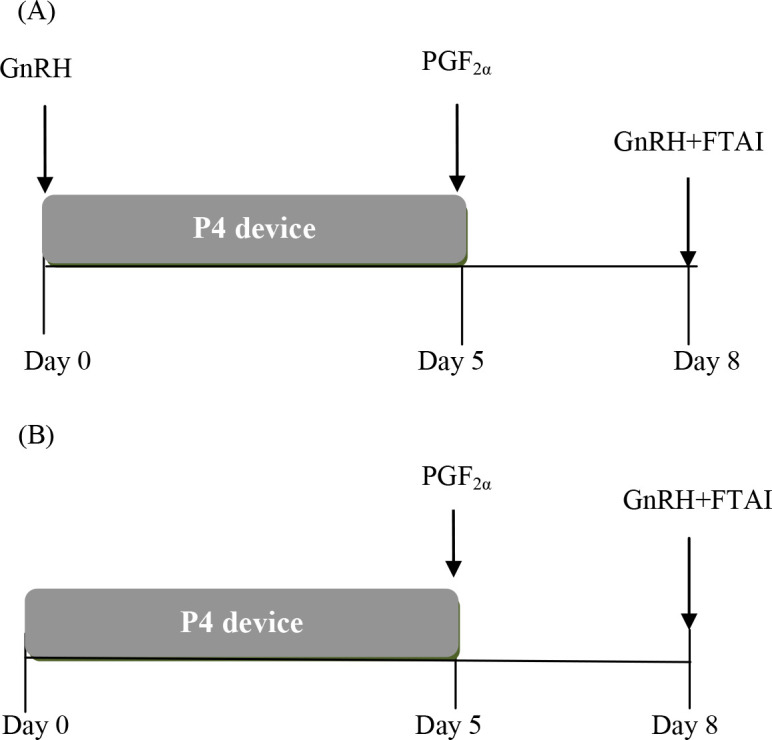
Two protocols for FTAI in beef cattle using GnRH. (A) 5-day Cosynch+P4 protocol. The interval from P4 device removal to FTAI is 66-72 h in heifers and 72 h in cows. If tail-paint or patches are used for estrus detection, FTAI begins at 60 h in all heifers with altered paint and those with the paint intact receive GnRH and are FTAI at 72 h. A second PGF_2_α administered at the same time of the device removal or 6 to 12 h later is recommended in cows and 400 IU of eCG may also be given in cows in postpartum anestrus. (B) Modified 5-day GnRH+P4 protocol. GnRH is not administered at P4-device insertion and only one PGF_2_α is required on Day 5. The recommended intervals from P4 device removal to FTAI are similar to those described previously.

The 5-day Co-Synch+P4 protocol has also been tested in heifers (Day, 2015), with some modifications introduced; for example, Colazo and Ambrose (2011) and Cruppe *et al*. (2014) showed that P/AI did not differ in heifers that did not receive GnRH at the time of insertion of a P4 device. The important issue with not administering the first GnRH is that a single injection of PGF_2α_ is all that is required. The two alternative 5-day Co-Synch+P4 protocols are depicted in Fig. 1.

Controversy still exists concerning the necessity of using one or two doses of PGF_2α_, when the first GnRH is administered with no reported differences (Kasimanickam *et al*., 2014) and higher P/AI when two doses of PGF_2α_ were used with intervals between 6 to 24 h (Day, 2015; Peterson *et al*., 2011). In relation to the optimal timing of FTAI, Kasimanickam *et al*. (2012) reported higher P/AI with heifers inseminated at 56 h after device removal than those inseminated at 72 h and Day (2015) suggested FTAI 60 to 66 h after P4 device removal or insemination 12 h after estrus using tail-patches or tail-paint and FTAI/GnRH to all those not in heat by 72 h. Certainly, expression of estrus has been shown to influence P/AI in cows (Richarson *et al*., 2016) and Colazo *et al*. (2017) have reported similar findings in heifers inseminated with sexed-sorted semen; suggesting the possibility of splitting the insemination based on estrus expression (i.e., delaying the insemination in those animals not showing estrus by the time of FTAI).

### Extending the proestrus period in estradiol/P4 based protocols

We have recently conducted a series of experiments to evaluate an estradiol/P4-based protocol with a prolonged proestrus interval, which has been named J-Synch (de la Mata and Bó, 2012). The treatment consists of the administration of 2 mg EB at the time of insertion of a P4 device that is removed 6 days later. A single dose of PGF_2α_ is given at device removal, and animals receive GnRH at the time of FTAI, 72 h later (Day 9).

A comparison of follicular and luteal dynamics between heifers treated with the 6-day J-Synch protocol and the conventional 7-day estradiol/P4-based protocol in which ECP was given at device removal (Day 7) showed that heifers in the J-Synch group ovulated 28 h later (93.7 ± 12.9 h after device removal; P < 0.05) than those in the conventional treatment (65.0 ± 13.7 h after device removal). Although the diameter of the preovulatory follicle did not differ between groups, the growth rate of the dominant follicle from P4 device removal to ovulation was greater in heifers in the J- Synch group (1.3 ± 0.4 mm/day) than those in the conventional group (1.0 ± 0.4; P < 0.05). Furthermore, serum P4 concentrations on Days 6 to 12 after ovulation were greater in heifers in the J-Synch group than in those in the conventional group (P < 0.05). Immunohistochemistry and real-time PCR of biopsies taken on Day 6 after ovulation suggested that heifers with the prolonged proestrus (J-Synch) had a more mature uterine environment for embryo development. This notion was based on lower (P < 0.05) staining intensity of the endometrial P4 receptors (PGR) in the intercaruncular uterine stroma and a tendency (P < 0.08) for lower PR mRNA and IGF_1_ mRNA in heifers treated with the J-Synch protocol than in those treated with the conventional protocol (de la Mata *et al*., 2018).

Several field trials were conducted to compare P/AI in heifers treated with the J-Synch protocol or the conventional estradiol/P4-based protocol (reviewed in Bó *et al*., 2016). In this series of experiments heifers in the J-Synch group were FTAI at the time of GnRH administration (i.e. 72 h after P4 device removal), whereas those in the conventional group were FTAI 54 h after device removal and ECP treatment. Although in the first experiment performed during the winter with heifers losing weight, P/AI was lower in heifers in the J- Synch group, in two experiments performed in the spring, with heifers gaining weight, the cumulative P/AI were higher in those treated with the J-Synch protocol than in those treated with the conventional estradiol/P4- based protocol. The contradictory results in the previous experiments was attributed to lower estradiol concentrations in the heifers that were losing weight in the winter compared to those gaining weight in the spring (Perry, 2017a). As it was mentioned earlier, high estradiol concentrations in the proestrous period have been associated with a more appropriate uterine environment, higher luteal phase P4 concentrations and a lower incidence of embryo loss (Perry, 2017b). Therefore, the administration of ECP in the heifers in the conventional protocol in the winter may have higher P/AI through higher exposure to estradiol and the ovulation of smaller follicles (Jinks *et al*., 2013). Conversely, the heifers treated in the spring while gaining weight may have had larger estradiol-producing follicles, thus no additional estradiol was needed to achieve high fertility (Perry, 2017a).

In order to avoid the confounding effects of nutrition on fertility in the J-Synch protocol, a study was designed to evaluate the effect of adding 300 IU of eCG at device removal to stimulate the growth of the dominant follicle on P/AI (Bó *et al*., 2016). The addition of 300 IU eCG at the time of P4 device removal resulted in higher P/AI (57.1%; 739/1295) as compared to those that did not receive eCG (53.1%; 692/1303). In another experiment, all heifers received 300 IU eCG at device removal but half were treated with the J-Synch protocol and the other half with the conventional estradiol/P4 based protocol with ECP at device removal. Again, P/AI was significantly higher in the J-Synch group (56.1%; 631/1,125) than in the conventional treatment group (50.7%; 620/1,224). The J-Synch protocol was also tested in 945 recipients receiving *in vitro*-produced embryos (Menchaca *et al*., 2015). Pregnancy rate following embryo transfer 7 or 8 d after GnRH (J-Synch + 400 IU eCG) or 9 or 10 d after ECP (conventional + 400 IU eCG) was higher (P < 0.01) in recipients synchronized with the J-Synch protocol (49.3%) than the conventional estradiol/P4-based protocol (40.9%). In conclusion, the strategy for extending proestrus significantly improved fertility in *Bos taurus* heifers. This improvement was considered to be due to a more competent CL that produced greater P4 concentrations in the ensuing luteal phase after AI and a uterine environment that may favor embryo development.

Although, more research is required in *Bos indicus* heifers, in one study, P/AI did not differ in Nelore and Nelore crossbred heifers treated with the J- Synch protocol or with the conventional protocol (both with 200 IU eCG at device removal; Motta *et al*., 2016). A similar approach of a prolonged proestrus and FTAI at 72 h after device removal, but by giving 1 mg EB 36 h after device removal instead of GnRH at the time of FTAI was evaluated in Brahman heifers (Edwards *et al*., 2015). P/AI was significantly higher in heifers treated with the prolonged proestrus protocol than those treated with the conventional 8-day protocol in one farm, but no differences were detected in two other farms.

### Proestrus length, estrus expression and GnRH treatments

An experiment was designed to evaluate the effect of the length of proestrus (i.e. interval from P4 device removal to GnRH and FTAI) on fertility in heifers (Núñez-Olivera *et al*., 2016). Angus and Angus crossbred heifers (n = 911) received a P4 releasing device containing 0.5 g P4 (DIB 0.5, Zoetis, Argentina) and 2 mg of EB (Gonadiol, Zoetis) on Day 0. At the time of P4 device removal (Day 6), 500 μg of cloprostenol (Ciclase DL, Zoetis) and 300 IU of eCG (Novormon, Zoetis) were administered IM. Heifers were then allocated in three groups to receive GnRH (100 µg gonadorelin acetate; Gonasyn GDR, Zoetis) and FTAI at 48, 60 or 72 h later. The diameter of the largest follicle (measured by ultrasonography) and estrus expression using tail-paint were recorded in a subset of heifers (n = 525) at the time of FTAI. Results are shown in Table 1. The largest follicle was smaller (P < 0.05) when GnRH/FTAI was performed at 48 h compared with 60 or 72 h. In addition, more heifers tended to display estrus by 72 h (P < 0.1) than 48 or 60 h. Although the overall P/AI tended to be greater (P < 0.1) in heifers inseminated at 72 h than at 48 or 60 h, P/AI was significantly greater (P < 0.05) among cycling heifers (i.e. with a CL on Day 0) in those FTAI at 72 h than in those FTAI at 48 or 60. In non-cycling heifers, P/AI did not differ among groups (58.9%, 247/419). Among the heifers showing estrus at the time of FTAI, P/AI was higher (P < 0.05) in those FTAI at 72 h (70.1%, 96/137) than in those FTAI at 60 h (56.7%, 68/120; P < 0.05), while 48 h was intermediate (63.9%, 78/122).

Based on the findings of the previous studies, it was proposed that heifers that manifest estrus earlier could be inseminated earlier without affecting P/AI, but it was needed to determine the optimum time for FTAI in those not showing estrus. To answer this question, 1,283 Angus and Hereford crossbred heifers were treated with the J-Synch protocol as described above and all heifers were tail-painted at P4 device removal. Heifers received GnRH/FTAI at either 60 or 72 h, regardless of paint removal. P/AI was higher in those that showed estrus prior to FTAI than in those that did not regardless of insemination time (53.6%, 542/1,012 vs. 45.0%, 122/271, respectively, P < 0.05). The P/AI in heifers that were in estrus by 60 h was similar whether the FTAI/GnRH was performed at 60 or 72 h. However, in those not showing estrus, P/AI was higher when the FTAI/GnRH was performed at 72 h (52%, 45/143) than at 60 h (37%, 47/128). The practical implication of this result is that when large herds are synchronized (i.e. 400 to 500 head), the device could be removed in the afternoon of Day 6 and FTAI begins at 60 h (Day 9 AM) in all heifers with altered paint as they come through the chute; those with the paint intact could be separated off to receive GnRH/FTAI in the afternoon of Day 9 (i.e. around or after 72 h).

**Figure 2:**
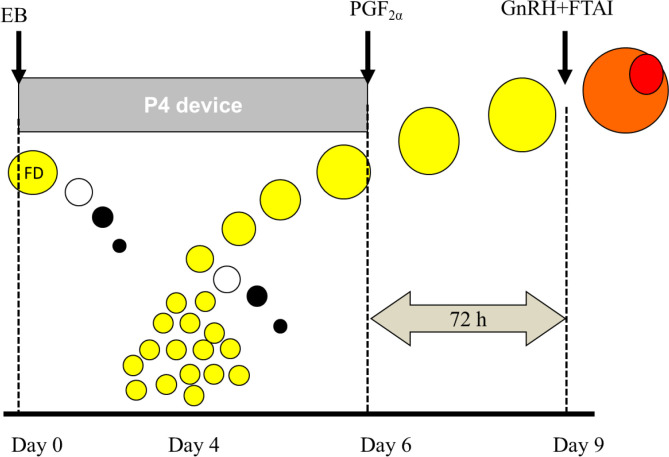
Estradiol/P4-based protocol with prolonged proestrus (J-Synch) in beef heifers. For FTAI without estrus detection, heifers receive GnRH and are inseminated 66-72 h after P4-device removal. If tail-paint or patches are used for estrus detection, FTAI begins at 60 h in all heifers with altered paint and those with the paint intact receive GnRH and are FTAI at 72 h. eCG (300 IU in heifers and 400 IU in cows) may also be given in those animals in anestrus.

The second question that was raised was the necessity to administer GnRH in those heifers that had already shown estrus prior to FTAI. An experiment was performed with 1,879 Angus heifers that were treated similarly to the previous study. All heifers displaying estrus at 60 h (85%, 1594/1879) were FTAI at that time, but GnRH was administered to only half. The heifers not displaying estrus at 60 h received GnRH/FTAI at 72 h. P/AI in the heifers that had manifested estrus by 60 h did not differ whether they received or did not receive GnRH (56.2% 451/802, vs. 58.6% 464/792, respectively), but P/AI was higher than in those not showing estrus by 60 h and receiving FTAI/GnRH at 72 h (40.4%, 15/285; P < 0.05). Therefore, with the use of tail-paint the cost of treatment could be reduced by giving GnRH to only the 25-30% of the heifers not showing estrus. The recommended protocol for FTAI is shown in Fig. 2.

Most of the experiments with the J-Synch protocol were performed in heifers and more information is needed about the performance of this protocol in lactating beef cows. Preliminary information using the J-Synch in non-lactating cows (i.e. after early weaning at 60 days postpartum) indicated P/AI were comparable to those reported in heifers (62.5%; 1,188/1,900), with higher P/AI in cows showing estrus by the time of FTAI (Menchaca *et al*., 2017). As with heifers, it is possible to avoid the use of GnRH at the time of the FTAI if tail-paint is used to determine the expression of estrus.

Only one study has been performed with the J- Synch protocol in suckled beef cows, with lower P/AI than in cows treated with the conventional estradiol/P4- based 7-day protocol (Bó *et al*., 2017), raising questions about the optimal period of P4 device insertion (6 or 7 days), the interval from device removal to ovulation (to determine the time for FTAI) and the requirement of ECP or GnRH to induce ovulation. More studies are underway to answer these questions.

**Table 1:** Diameter of the largest follicle, estrus expression and P/AI in beef heifers synchronized with the J-Synch protocol and received GnRH and were fixed-time AI at different intervals from the removal of the P4 releasing device.

	Follicular diameter at FTAI (mm)	Heifers in estrus at FTAI (%)	P/AI in cycling heifers^*^ (%)	Overall P/AI (%)
GnRH/FTAI 48 h	12.2 ± 0.1^a^	68.2%^a^	67.7%^a^	63.6%^c^
	(n = 179)	(122/179)	(107/158)	(196/308)
GnRH/FTAI 60 h	12.8 ± 0.1^b^	71.4%^a^	68.3%^a^	63.1%^c^
	(n = 168)	(120/168)	(110/161)	(183/290)
GnRH/FTAI 72 h	12.9 ± 0.2^b^	77.0%^a^	77.5%^b^	70.0%^d^
	(n = 178)	(137/178)	(134/173)	(219/313)

ab Denotes significant (P < 0.05) differences between treatment groups.

cd Denotes a tendency (P < 0.1) for higher overall P/AI in heifers inseminated at 72 h.

* Heifers with a CL detected by ultrasonography at the time of P4 device insertion.

## Protocols for fixed-time AI using sexed-sorted semen

The use of sexed-sorted semen has created great interest in the insemination of dairy heifers over the past 20 years; however, the widespread use of sexed semen was somewhat limited because fertility was compromised, especially with FTAI (DeJarnette *et al*., 2011). However, the sorting procedure has been improved and a new product called SexedULTRA^TM^, with the option of increasing the number of sperm from 2.1x10^6^ to 4x10^6^ sperm per straw has been launched (Vishwanath, 2015).

**Figure 3:**
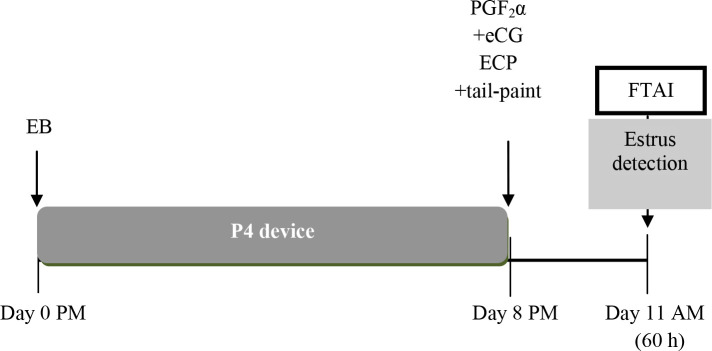
Conventional estradiol/P4-based protocol for FTAI with sexed-sorted semen. Tail-paint or patches are used to detect those animals in estrus 60 h after P4-device removal. Animals in estrus by 60 h are inseminated with sexed-sorted semen whereas those animals not in estrus by 60 h receive GnRH and are inseminated at the same time with sexed-sorted or non-sexed semen.

A series of experiments were designed to determine P/AI using modifications of the existing estradiol/P4-based protocols for sexed semen. Two experiments were carried out using suckled Nelore cows that received the conventional protocol for FTAI as follows: 2 mg EB + P4 device on Day 0, removal of the device and PGF_2α_ + 300 IU of eCG + 1 mg of ECP on Day 8 and FTAI 60 h after P4 device removal (Baruselli *et al*., 2017b). In the first experiment, 796 cows were inseminated with female sexed-selected semen and non- sexed (Control) semen from three different Nelore bulls. The experimental groups were: 1) non-sexed semen with 20x10^6^ sperm per dose; 2) Legacy (previous method of sexing), sexed semen with 2.1x10^6^ sperm per dose; 3) SexedULTRA with 2.1x10^6^ sperm per dose and 4) SexedULTRA 4.0, sexed semen with 4.0x10^6^ sperm per dose. No differences were detected between sires (P = 0.15). Although P/AI was highest (P < 0.05) in cows in the non-sexed semen group (56%, 112/199), cows in the SexedULTRA 4.0 group had higher P/AI (43%, 86/200) than those in the Legacy 2.1 group (28%, 58/206). In cows inseminated with the SexedULTRA 2.1 semen P/AI was intermediate (38%, 72/191) and not different than the other groups inseminated with sexed semen. In the second experiment, 613 cows were treated as those in the first experiment and inseminated with semen from three Angus sires. The experimental groups were 1) non-sexed semen (20x10^6^ sperm); 2) SexedULTRA 4.0 and 3) SexedULTRA Pure 4.0 (dead sperm were removed). A subgroup of 431 cows was tail-painted for estrus determination at the time of FTAI. P/AI among sires did not differ (P = 0.12). Overall P/AI was not different among groups (non-sexed semen: 51.2%, 107/209; SexedULTRA 4.0: 42.0%; 84/200 and SexedULTRA Pure 43.1%; 88 / 204; P = 0.10; Baruselli *et al*., 2017b). Furthermore, in the subgroup of animals in which estrus was recorded there was a significant interaction between type of semen and expression of estrus (P < 0.01). In cows showing estrus, P/AI was not different between cows inseminated with sexed or non- sexed semen (Table 2). Conversely, in cows not showing estrus P/AI was higher (P < 0.05) for those inseminated with non-sexed semen and those inseminated with SexedULTRA Pure, whereas P/AI in those inseminated with SexedULTRA was intermediate and not different from the other two groups. The recommended protocol for FTAI with sexed-sorted semen is shown in Fig. 3.

**Figure 4:**
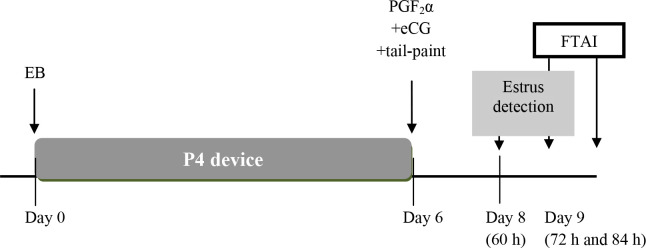
Alternative estradiol/P4 based protocol with prolonged proestrus for FTAI beef heifers with sexed-sorted semen. Tail-paint or patches are used to detect those heifers in estrus at 60 and 72 h after P4-device removal. Heifers in estrus at 60 and 72 h are inseminated at 72 h with sexed-sorted semen. Heifers not in estrus by 72 h receive GnRH and are inseminated at 84 h with sexed sorted or non-sexed semen.

Another study was carried out to evaluate P/AI in heifers treated with the J-Synch protocol and inseminated with SexedULTRA semen (Huguenine *et al*., 2017, unpublished). Angus and Angus crossbred beef heifers (n = 850) were treated with the J-Synch protocol with 300 IU eCG at P4 device removal (Day 6) and were tail-painted for estrus determination. The heifers with the paint-rubbed off at 60 or 72 h after device removal were randomly subdivided into two subgroups to be inseminated at 72 h with female SexedULTRA 4.0 semen or with non-sexed semen from the same four Angus sires. Heifers that did not showed estrus by 72 h received GnRH at that time and were inseminated at 84 h with the same two types of semen. The protocol is shown in Fig. 4 and the results are shown in Table 3. There were 72.7% (618/850) of heifers in estrus at 60 and 72 h and the overall P/AI was 54.0% (459/850) regardless of expression of estrus. There was a significant (P < 0.01) effect of semen type (Table 3), time of AI and sire on P/AI, but no interactions. P/AI were 64.1% (223/348) for heifers in estrus at 60 h and inseminated at 72 h, 51.8% (140/270) for those in estrus and inseminated at 72 h, and 40.9% (95/232) for those not in estrus by 72 h and inseminated at 84 h after P4 device removal. Regarding sires, P/AI ranged from 40.5% to 67.8% with non-sexed semen and 26.5% to 59.3% with SexedULTRA semen. In summary, protocols designed for FTAI in cows and heifers can be adapted for sexed-sorted semen. Although, P/AI are lower than those obtained with non- sexed semen, delaying the time of AI or limiting the AI to those animals showing estrus would result in P/AI between 40 and 50% or even higher.

**Table 2:** Pregnancy rates in lactating Nelore cows inseminated with sexed-sorted (SexedULTRA^TM^) or non-sexed semen according to the expression estrus (reading of paint-stick) and time of insemination (AI) after the removal of the progesterone releasing device.

	n	Estrus 60 h AI 60 h	No estrus at 60 h AI 60 h	Total
SexedULTRA (4x10^6^ sperm)	138	50/94 (53.1%)	14/44 (31.8%)^ab^	64/138 (46.4%)
SexedULTRA Pure (4x10^6^ sperm)	144	54/102 (52.9%)	9/42 (21.4%)^bc^	63/144 (43.8%)
Non-sexed (20x10^6^ sperm)	149	57/110 (51.8%)	18/39 (46.2%)^c^	75/149 (50.3%)

abc Denotes differences in P/AI between sexed-sorted or non-sexed semen in cows not showing estrus (P < 0.05).

**Table 3:** Pregnancy rates in Angus heifers inseminated with sexed-sorted (SexedULTRA^TM^) or non-sexed semen according to the expression estrus (reading of tail-paint) and time of insemination (AI) after the removal of the progesterone releasing device.

	n	Estrus 60 h AI 72 h	Estrus 72 h AI 72 h	No estrus at 60 or 72 h AI 84 h	Total
SexedULTRA (4x10^6^ sperm)	426	104/176 (59.0%)	61/134 (45.5%)^a^	45/116 (38.8%)	210/426 (49.3%)^a^
Non-sexed (25x10^6^ sperm)	424	119/172 (69.2%)	79/136 (58.0%)^b^	49/116 (42.2%)	247/424 (58.3%)^b^

ab Denotes differences in P/AI between sexed-sorted or non-sexed semen (P < 0.05).

## Re-synchronization treatments

With the advent of FTAI in beef herds, producers have been seeking strategies that can be implemented to obtain the highest possible number of cows pregnant through AI early in the breeding season. Several approaches have been developed over the years; however, most require estrus observations or an interval of approximately 40 days between the first and second FTAI (reviewed in Bó *et al*., 2016 and Baruselli *et al*., 2017a). In order to be able to inseminate non-pregnant cows as early as possible, re-synchronization treatments must start earlier than pregnancy diagnosis. One protocol was developed in which a re-used device impregnated with 1 g of P4 (i.e. previously used in the first synchronization) was re-inserted on Days 14-16 after the first AI and is removed on Day 23 at the time that GnRH was administered. Pregnancy diagnosis is performed on Day 30 and those that are found to be non-pregnant receive PGF_2α_ combined with either 0.5 mg ECP at that time or simply GnRH at the time of FTAI on Day 32. P/AI for the first and second FTAI and the overall P/AI in a field trial involving 6,431 beef cows and heifers were 57%, 51% and 79%, respectively (Bó *et al*., 2016).

Two new protocols for re-synchronization are called Resynch 22 and Resynch 14 (Baruselli *et al*., 2017a). In the Resynch 22, cows receive 2 mg EB (Pessoa *et al*., 2015) and heifers 1 mg EB (Sá Filho *et al*., 2014) at P4 device insertion on Day 22. Pregnancy diagnosis is performed at device removal (Day 30) and non-pregnant animals also receive PGF_2α_ and ECP and are inseminated on Day 32. The Resynch 14 protocol involves the use of color Doppler ultrasonography for the detection of pregnancy based on the vascularization and size of the CL on Day 22 after the first AI (Siqueira *et al*., 2013; Pugliesi *et al*., 2014, 2017). For Resynch 14 the initial treatment starts 14 days after FTAI with the re-insertion of a re-used device and the administration of 100 mg P4 IM (Rezende *et al*., 2016) to avoid the possible luteolytic effect of EB (Vieira *et al*., 2014). The new wave emerges 3.0 ± 0.7 days after P4 administration in Nelore cows (Rezende *et al*., 2016); thus cows are scanned with Doppler ultrasonography for pregnancy at device removal (Day 22) and non-pregnant animals receive PGF_2α_ and ECP and are inseminated on Day 24. In a recent study, similar P/AI were observed for Resynch 22 and Resynch 14 groups following the first FTAI (48% vs 53%; P = 0.57) and re- synchronization (56% vs 51%; P = 0.37), respectively. However, the Resynch 14 reduced the interval between FTAI, which resulted in a 21-day P/AI of 87.5% compared to 66% with the Resynch 22 (Penteado *et al*., 2016). In a more recent field trail, the use of three consecutive FTAI with the Resynch 22 protocol had a similar overall pregnancy rate (87.8%, 663/755) as that achieved using clean-up bulls after two FTAI using Resynch 22 (87.7%, 263/300) and greater pregnancy rate than one FTAI followed by bull exposure (77.1%, 347/450; Crepaldi *et al*., 2017). In conclusion, with the existing re-synchronization programs it is now possible to breed beef cows exclusively with FTAI, eliminating the need for estrus detection and clean-up bulls.

## Summary and conclusions

Protocols for FTAI have allowed the widespread use AI in South-America, with the possibility of obtaining P/AI of 50% or more with a single insemination. The addition of eCG at P4-device removal to stimulate the growth of the ovulatory follicle has been especially useful in increasing P/AI in cows experiencing postpartum anestrus. New treatment protocols that do not require the use of estradiol or GnRH shortly after P4 device removal and allow for a longer proestrus period prior to insemination are alternatives for increasing fertility to FTAI; however, the animals must be in optimal nutritional conditions to achieve high P/AI. Furthermore, FTAI treatments combined with estrus detection using tail-paint or heat- detection patches permits the use of sexed-sorted semen with acceptable P/AI. Finally, early pregnancy diagnosis with ultrasonography can be easily implemented in beef herds in order to perform sequential FTAI without estrus detection, resulting in pregnancy rates that are similar or higher than obtained with clean-up bulls, maximizing the use of the improved genetics through AI.
